# Electrospun Composites of Chitosan with Cerium Oxide Nanoparticles for Wound Healing Applications: Characterization and Biocompatibility Evaluation In Vitro and In Vivo

**DOI:** 10.3390/polym16131787

**Published:** 2024-06-25

**Authors:** Valentina A. Petrova, Daria N. Poshina, Alexey S. Golovkin, Alexander I. Mishanin, Sergei G. Zhuravskii, Galina Y. Yukina, Maria Y. Naumenko, Elena G. Sukhorukova, Nikita A. Savin, Alexander S. Erofeev, Iosif V. Gofman, Elena M. Ivan’kova, Natallia V. Dubashynskaya, Alexander V. Yakimansky, Yury A. Skorik

**Affiliations:** 1 Institute of Macromolecular Compounds of the Russian Academy of Sciences, Bolshoi VO 31, 199004 St. Petersburg, Russia; valentina_petrova_49@mail.ru (V.A.P.); poschin@yandex.ru (D.N.P.); gofman@imc.macro.ru (I.V.G.); ivelen@mail.ru (E.M.I.); dubashinskaya@gmail.com (N.V.D.); yak@hq.macro.ru (A.V.Y.); 2 Almazov National Medical Research Centre, Akkuratova 2, 197341 St. Petersburg, Russia; golovkin_a@mail.ru (A.S.G.); mishaninssma@yandex.ru (A.I.M.); 3 Hearing and Speech Laboratory, Pavlov First Saint Petersburg State Medical University, L’va Tolstogo 6-8, 197022 St. Petersburg, Russia; s.jour@mail.ru (S.G.Z.); naumenkomyu@gmail.com (M.Y.N.); 4 Laboratory of Pathomorphology, Pavlov First Saint Petersburg State Medical University, L’va Tolstogo 6-8, 197022 St. Petersburg, Russia; pipson@inbox.ru (G.Y.Y.); len48@inbox.ru (E.G.S.); 5 Laboratory of Biophysics, National University of Science and Technology “MISIS”, Leninsky 4, 119049 Moscow, Russia; nsavin99@mail.ru (N.A.S.); erofeev.as@misis.ru (A.S.E.)

**Keywords:** cerium oxide, chitosan, electrospinning, wound dressing, tissue engineering

## Abstract

Cerium oxide nanoparticles (CeONPs), as part of tissue regeneration matrices, can protect cells from reactive oxygen species and oxidative stress. In addition, they can influence the properties of the scaffold, including its electrospinnability and mechanical strength. In this work, we prepared electrospun fiber mats from a chitosan and polyethylene oxide blend (CS-PEO) with the addition of ceria nanoparticles (CS-PEO-CeONP). The addition of CeONPs resulted in a smaller fiber diameter and higher swelling compared to CS-PEO fiber mats. CeONP-modified fiber mats also had a higher Young’s modulus due to the reinforcing effect of the nanoparticles. Both mats had comparable adhesion and cytocompatibility to mesenchymal stem cells, which had a more rounded morphology on CS-PEO-CeONP compared to elongated cells on the CS-PEO mats. Biocompatibility in an in vivo rat model showed no acute toxicity, no septic or allergic inflammation, and no rough scar tissue formation. The degradation of both mats passed the stage of matrix swelling. CS-PEO-CeONP showed significantly slower biodegradation, with most of the matrix remaining in the tissue after 90 days. The reactive inflammation was aseptic in nature with the involvement of multinucleated foreign-body type giant cells and was significantly reduced by day 90. CeONPs induced the formation of the implant’s connective tissue capsule. Thus, the introduction of CeONPs influenced the physicochemical properties and biological activity of CS-PEO nanofiber mats.

## 1. Introduction

The development of nanofibrous materials by electrospinning for use as 3D scaffolds in tissue engineering has attracted great interest from researchers. One of the challenges in this field is to understand the relationship between the chemical structure of polymers, the properties of electrospinning solutions, the architecture of the resulting nanofibrous materials, and their biocompatibility. Electrospun mats with high porosity and large internal surface area are perfect scaffolds for tissue engineering because they mimic the properties of collagen fibers of natural extracellular matrices [1,2]. Compared to macroporous and microfiber scaffolds, nanofibers provide maximum contact points for cell receptors, creating a 3D effect for cell adhesion and proliferation [3]. The mechanical strength of electrospun mats is higher than that of hydrogels [4].

Natural polysaccharides are particularly promising materials for tissue engineering [5] because they are nontoxic, biodegradable, and biocompatible. Among natural polysaccharides, most publications refer to chitosan (CS) [6,7] and various composite materials involving CS [8,9]. However, electrospinning CS has many difficulties associated with the high conductivity and high surface tension of CS solutions, as well as strong intermolecular interactions, resulting in unstable electrospinning [10].

One of the important tasks in tissue engineering is to protect cells from oxidative stress. For this purpose, materials that can protect cells from reactive oxygen species have been investigated, such as cerium oxide nanoparticles (CeONPs) [11,12]. When incorporated into polymer matrices, CeONPs can regulate the physicochemical properties of materials and influence their interactions with biosystems [13]. Previously, in a series of works on composites containing CeONPs, we have established the effect of CeONPs on the physicochemical properties of polymer composites, as well as the effect of the polymer component on the activity of CeONPs against mesenchymal stem cells. The optimal compositions for obtaining polymer composites with improved bioactivity were also determined [14,15,16]. The introduction of CeONPs was found to alter the structural organization of CS films [14] and CS–bacterial cellulose composites [15,16], affecting their morphology and increasing swelling. The nanocomposites showed increased Young’s modulus and good strength properties, indicating strong intermolecular interactions between matrix and nanofiller. All composites were found to be biocompatible. CeONPs increased the proliferation rate of cells by enhancing their migration from spheroid colonies. The optimal level of CeONPs in these studies was found to be 8%.

The aim of this study is to obtain an electrospun mat that combines the valuable properties of the biocompatible CS polysaccharide with those of the active CeONP nanofiller. This approach will allow to tune the structural and physicochemical properties of electrospun scaffolds, as well as their bioreactivity, and further develop an interesting scientific and applied task to improve tissue engineering scaffolds and wound dressings.

## 2. Materials and Methods

### 2.1. Materials

The crab CS (Bioprogress, Shchelkovo, Russia) used in this study had the following characteristics: a characteristic viscosity [η] of 2.56 dL/g, a viscosity average molecular weight (Mη) of 6.0 × 10^4^ (determined using an Ubbelohde capillary viscometer at 20 °C and calculated from the Mark-Houwink equation [η] = 3.41 × 10^−3^ Mη^1.02^ [17]), and degree of deacetylation of 82% (determined by ^1^H NMR spectroscopy). Other materials and reagents included polyethylene oxide (PEO) with molecular weight of 9.0 × 10^5^ (Sigma Aldrich, St. Louis, MO, USA).

CS-stabilized CeONPs were prepared according to the previously described procedures [14,18]. Briefly, 1% aqueous dispersion of citrate-stabilized CeO_2_ was mixed with a diluted 0.1% CS solution in 1% acetic acid using an IL 100-6 (Russia) ultrasonic homogenizer within 10 min; the mass ratio of the components CeONP:CS was 1:1.

### 2.2. Electrospinning

The polymer concentrations in the electrospinning solutions were chosen experimentally to ensure uniform spinning; the optimal concentration of CS was found to be 3%. CS was first suspended in water with vigorous stirring for several hours; then, glacial acetic acid was added with continuous stirring. When the CS was completely dissolved, a 3% PEO solution in water was added. The final concentrations were as follows: 3% CS and 0.3% PEO in 70% acetic acid (CS-PEO solution). The electrospinning solution with the addition of CeONPs (CS-PEO-CeONP) was prepared by adding the CS-stabilized CeONPs to the CS-PEO solution with continuous vigorous stirring. The final concentration of CeONPs in the electrospinning solution was 8% with respect to CS.

Electrospinning was performed using a non-capillary Nanospider NS Lab 500 machine (Elmarco, Liberec, Czech Republic). The electrospinning voltage was varied from 50 to 65 kV to obtain stable and uniform solution jets. The rotation speed of the spinning electrode was 10 min^−1^ and the distance between the electrodes was 24 cm. The fibers were collected on a paper substrate. Two electrospun nanofiber mats were obtained: CS-PEO and CS-PEO-CeONP. After electrospinning, the samples were kept at room temperature for 5 days and then heated at 80 °C for 3 h.

### 2.3. General Methods

The resulting mats were characterized by scanning electron microscopy (SEM) using a SUPRA setup (Carl Zeiss, Oberkochen, Germany) and by wide-angle X-ray scattering (WAXS) using a Bruker D8 DISCOVER X-ray diffractometer with CuKα radiation (Bruker, Karlsruhe, Germany). SEM images were obtained using a secondary electron detector and a backscattered electron detector. 

The equilibrium swelling capacity of the electrospun mats in water and physiological solution (0.9% NaCl) was determined by the gravimetric method by soaking the electrospun mat in the corresponding solution for 24 h. The degree of swelling was defined as follows:

Swelling (g/g) = (W_s_ − W_d_)/W_d_,

where W_d_ is the initial weight of the air-dried mat and W_s_ is the weight of the swollen mat.

Swollen fiber topography and local mechanical properties were performed using a scanning ion conductance microscope (SICM) from ICAPPIC (ICAPPIC Ltd., London, UK). Glass nanopipettes with a typical radius of 45–50 nm were prepared using a Laser Puller P-2000 (Sutter Instruments, Novato, CA, USA). Scanning was performed in non-contact hopping mode with adaptive resolution and maps of 5 × 5 or 10 × 10 μm. The speed of approach during imaging was set to 50 μm s^−1^. A non-contact topographic image was obtained at an ion current drop of 0.3% and a Young’s modulus image at a drop of 1 and 2%. Scanning was performed in Hanks’ balanced salt solution.

An AG-100kNX Plus setup (Shimadzu, Kyoto, Japan) operating in uniaxial extension mode was used to investigate the mechanical properties of the films. Strip-like samples (2 × 30 mm) were stretched at a rate of 20 mm/min at room temperature according to the requirements of ASTM D638 [19]. The stress–strain curves of the samples were recorded during the tests. Young’s modulus (E), yield stress (σ_y_), break stress (σ_b_), and elongation at break (ε_b_) were determined.

### 2.4. Culture of Multipotent Mesenchymal Stem Cells (MMSCs)

The cell adhesion properties of the materials were evaluated using MMSCs derived from visceral adipose tissue of healthy male Wistar rats. This study was approved by the Commission for the Control of Care and Use of Laboratory Animals of the Almazov National Medical Research Centre (protocol No. 21-12PZ#V3 dated 13 July 2021). This study was performed as described previously [8,14,16,20]. Briefly, cell culture was performed under standard conditions and co-cultured with rectangular test samples at a concentration of 50,000 cells/mL for 72 h. Cell culture carried out with coverslips under the same conditions was used as a control. 

The samples and coverslips were then fixed and stained with rhodamine-conjugated phalloidin (Thermo Fisher Scientific, Waltham, MA, USA) solution (1:500) and 4,6-diamidino-2-phenylindole (DAPI, 1:40,000) (Sigma-Aldrich, Co., St. Louis, MO, USA). Stained cells were visualized using an Axiovert inverted fluorescence microscope (Zeiss, Oberkochen, Germany). Images were captured using a Canon camera (Canon Europa N.V., Amstelveen, The Netherlands). Quantitative analysis of adherent cells was performed by examining 10 fields of view. Qualitative analysis was performed at both 10× and 40× magnification. Statistical processing of the data obtained was performed with GraphPad Prism 8 software (GraphPad Software Inc., San Diego, CA, USA) using the nonparametric Mann–Whitney U test.

### 2.5. In Vivo Studies

All procedures were performed in accordance with the Guide for the Care and Use of Laboratory Animals (NIH Publication No. 85-23, revised 1996) and the European Convention for the Protection of Vertebrate Animals used for Experimental and other Scientific Purposes. The study protocol was approved by the Institutional Animal Care and Use Committee of Pavlov First St. Petersburg State Medical University (protocol number PZ_21-02#Zhuravskii S.G. V3. 18 September 2023). All efforts were made to protect the animals and minimize their suffering during this study. The experiments complied with the ARRIVE guidelines https://arriveguidelines.org/ (accessed on 15 October 2023). 

Forty male Wistar rats (200–220 g, “Rappolovo” Nursery for Laboratory Animals, National Research Center Kurchatov Institute) were kept in a conventional vivarium (Pavlov First St. Petersburg State Medical University). Animals were divided into 4 groups: intact, sham-operated, implanted with CS-PEO mats, and implanted with CS-PEO-CeONP mats. The samples were cut into 10 × 10 mm pieces; the thickness of the pieces was approximately 60 μm. Sterilization was performed by soaking in 70% ethanol for 24 h. Prior to implantation, the samples were soaked in sterile 0.9% NaCl solution for 24 h and washed 3 times for 15 min in sterile 0.9% NaCl solution to remove residual ethanol.

Under aseptic conditions and general anesthesia (Zoletil and Xylazine), a 1 cm incision was made in the lumbar region to create an implant bed between the dermis and the sternolumbar fascia. After placing the samples in the implant pocket, the wound was closed with interrupted sutures using Prolene 6/0 suture material.

Animals were sacrificed by removal of vital organs under deep general anesthesia. Three rats were sacrificed at each observation time point. Skin samples were collected from the implantation zone for histologic analysis. Skin samples were fixed in formalin. They were then dehydrated through a graded series of ethanol according to a conventional protocol and embedded in paraffin to prepare a paraffin block. Sections of 5 μm were cut with an Accu-Cut SRM 200 microtome (Sakura, Torrance, CA, USA) and stained with hematoxylin and eosin (Biovitrum, St. Petersburg, Russia). Histologic microimages obtained on days 30, 60, and 90 after surgery were visually analyzed for thickness and structure of implanted matrix, type and location of cellular infiltration, and connective tissue growth. Microscopic analysis was performed on a Nikon Eclipse Ni light microscope (Nikon, Tokyo, Japan) equipped with a Nikon DS-Fi2 camera using NIS-Elements BR (v. 4.40) software.

## 3. Results 

### 3.1. SEM Morphology of the CS-PEO and CS-PEO-CeONP

**Figure 1:**
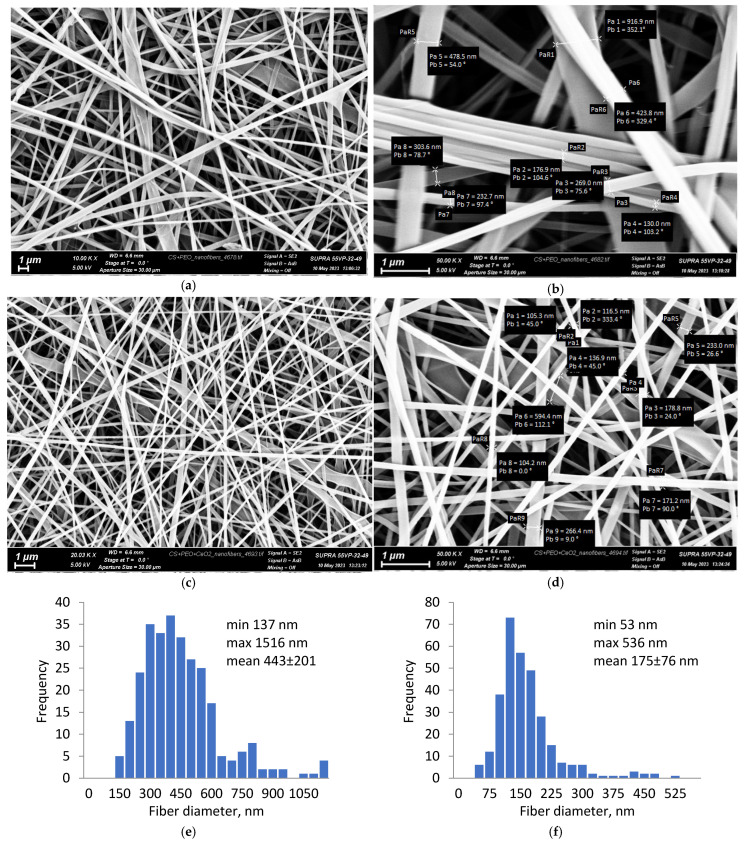
SEM images of the CS-PEO (**a**,**b**) and CS-PEO-CeONP (**c**,**d**) nanofibers and corresponding diameter distributions (**e**,**f**).

The electrospinning parameters were experimentally selected for each composite solution to ensure the stability of the electrospinning process and to obtain uniform nanofibers. The resulting electrospun mats had a thickness of 50–60 μm; they were heated at 80 °C for 3 h to convert them to an insoluble form. The SEM image of CS-PEO (Figure 1a,b) showed the uniform formation of nanofibers with an average diameter of 443 ± 201 nm (Figure 1e). The electrospinning of the composite solution with CeONPs also proceeded without defects (Figure 1c,d); the fiber diameter ranged from 53 to 536 nm with an average value of 175 ± 76 nm (Figure 1f). The resulting nanofiber mats were similar in morphology to the extracellular matrix of natural tissues and therefore could provide a suitable environment for stem cell culture [21].

### 3.2. XRD Structure 

**Figure 2:**
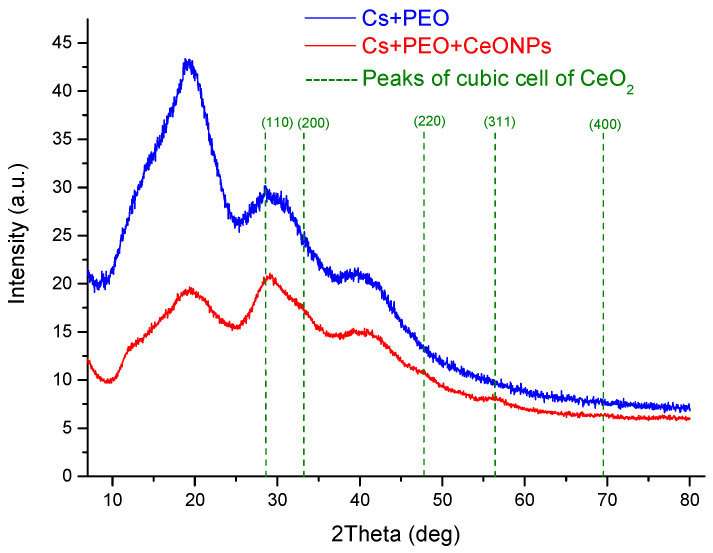
XRD profiles of CS-PEO and CS-PEO-CeONP electrospun mats.

The diffraction pattern of electrospun CS-PEO (Figure 2) had a broad reflex in the 2θ range of 15–23.7°, which is typical for the anhydrous CS polymorph (it has three strong reflexes: 2θ = 15°, 21.5°, 23.7°) [22,23]. The CS diffraction in nanofibers was also superimposed with PEO reflexes at 2θ 23 and 28°. The diffractogram of the CS-PEO-CeONP nonwoven shows, in addition to the above reflexes, ones characteristic of CeONPs. The faint reflections of CeONPs at 2θ of 28.7, 33.0, 47.5, and 57.0° correspond to CeO_2_ crystal planes (110), (200), (220), and (311), respectively (cubic crystal structure of fluorite): ICDD PDF map #34-394, data from National Institute of Standards and Technology, Gaithersburg, MD, USA) [16].

### 3.3. Mechanical Properties

**Figure 3:**
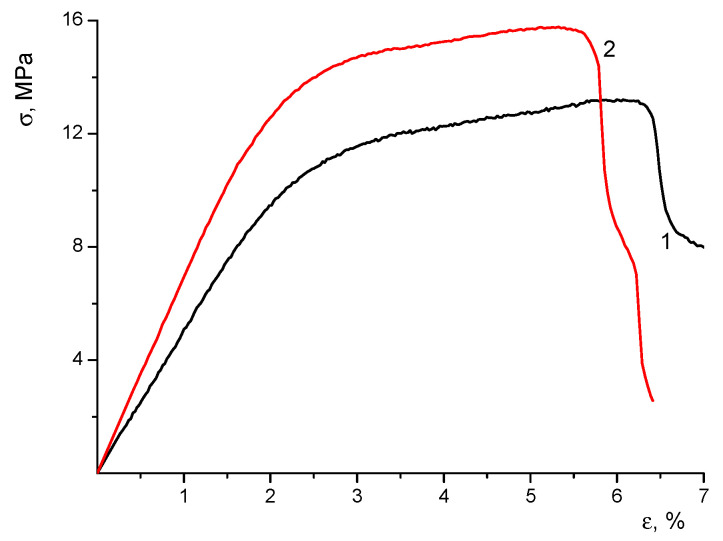
Stress–strain curves of CS-PEO (1) and CS-PEO-CeONPs (2) electrospun mats.

The mechanical properties of the materials studied are summarized in Table 1, and their stress–strain curves are shown in Figure 3. The character of deformation in all samples is common for electrospun materials; no necking was observed when the samples were stretched beyond the yield point. The ultimate deformation values were 5–6%, which are quite adequate for their application in biomedicine.

The introduction of CeONPs into the CS-PEO polymer matrix resulted in a significant increase in Young’s modulus (E, Table 1) and a small increase in the values of yield stress and break stress (σ_y_ and σ_b_, Table 1). An increase in the stiffness of the material caused by the introduction of CeONPs into the polymer matrix was a result of the formation of additional bonds between the polymer macromolecules and the nanoparticle surface [14]. At the same time, the formation of the nanocomposites resulted in a certain decrease in the ultimate deformation of the samples (ε_b_, Table 1). This effect is typical for the polymer-inorganic nanocomposites of different compositions and is related to the increase in material heterogeneity [24,25,26]. 

### 3.4. Swelling Properties 

The introduction of CeONPs contributed to an increase in the degree of swelling (Table 2), which is related to a rearrangement of the intermolecular bonds of the CeONP system. We have previously observed similar results for CS-based films containing CeONPs [14]. A lower swelling of CS in 0.9% NaCl solutions compared to pure water has been reported previously. This was suggested to occur due to an increase in osmotic pressure in the presence of salts [27] or due to the shielding of CS amino groups by counterions [28]. The nanofillers, which strongly interact with CS macromolecules, can also alter the swelling capacity in physiological solutions [29]. In our case, the addition of CeONPs significantly increased the swelling in water, but it also made the decrease in swelling caused by NaCl much more pronounced.

**Figure 4:**
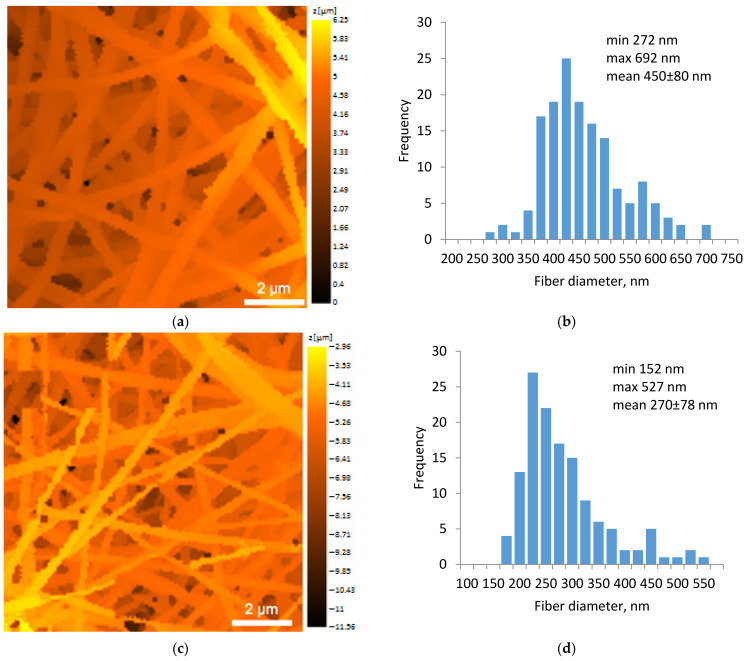

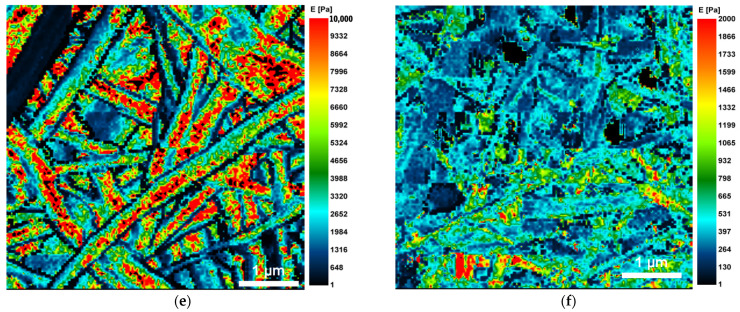
SICM images of the CS-PEO (**a**) and CS-PEO-CeONP (**c**) nanofibers swollen in Hanks’ balanced salt solution, as well as corresponding diameter distributions (**b**,**d**) and Young’s modulus distributions (**e**,**f**).

After 1 h of swelling, the mats retained their fiber morphology well (Figure 4a,c). The number of relatively thin fibers in the CS-PEO-CeONP mat was visibly higher. Incomplete swelling after 1 h allowed us to compare the swelling rate of the two fiber types over time. The diameter of the CS-PEO fibers remained virtually unchanged (Figure 4b), while the diameter of the CS-PEO-CeONP fibers increased more significantly, by approximately 150% (Figure 4d). These results were in good agreement with the equilibrium swelling results presented in Table 2.

Local mechanical properties measured via scanning ion conductance microscopy (SICM) showed that CS-PEO fibers are more difficult to deform compared to CS-PEO-CeONP fibers (Figure 4e,f). This may be related to the large average diameter as well as the CS-CeONP interactions and rearrangement of intramolecular bonds. This rearrangement may result in a less dense packing of macromolecules in the presence of CeONPs and a lower overall local stiffness of the CS-PEO-CeONP fibers [14,16].

### 3.5. Cytocompatibility on Multipotent Mesenchymal Stem Cells 

**Figure 5:**
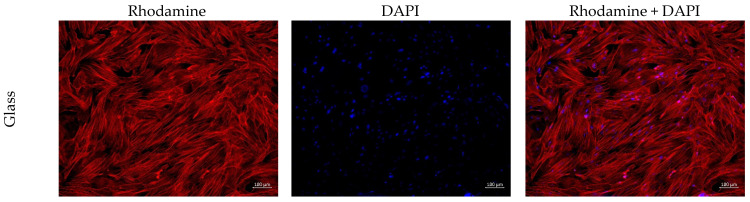

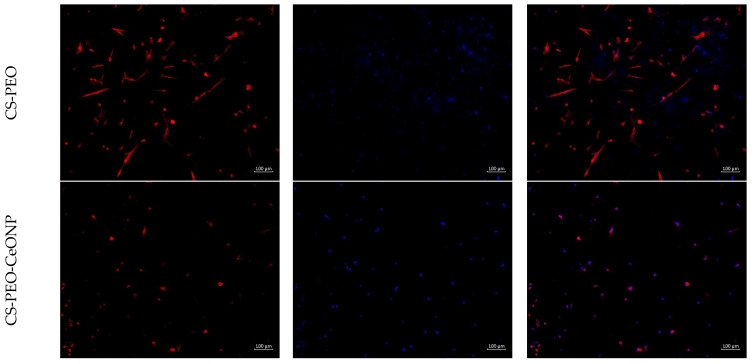
MMSCs adhered on the samples surface, ×100 magnification.

**Figure 6:**
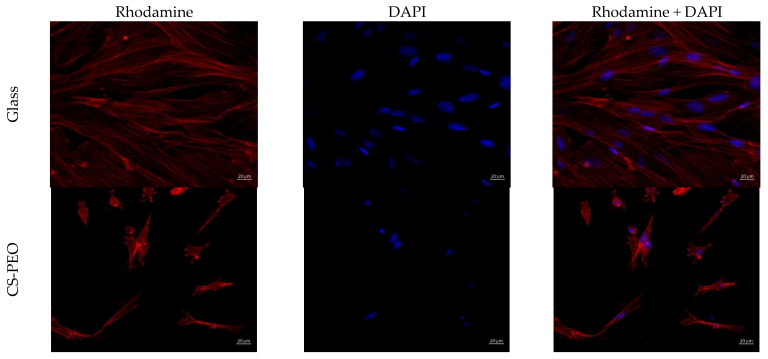

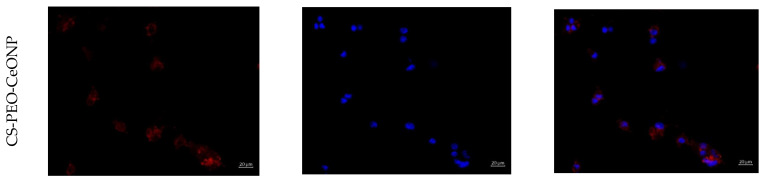
MMSC adhered on the samples surface, ×400 magnification.

A cell scaffold, fabricated as the electrospun mat, should provide an optimal environment for cell adhesion, as well as for proliferation, migration, and differentiation. There were no significant differences in the number of adherent cells between the two samples (Table 3). However, cell morphology was rather different (Table 3, Figure 5 and Figure 6).

Adhered cells on CS-PEO were numerous, and most of them had a typical elongated shape with longitudinal striations (Figure 5 and Figure 6). Cells tended to spread out over the material’s surface. However, some cells had a spherical shape, with protrusions demonstrating no adherence nor proliferation. On CS-PEO-CeONP, elongated cells with good proliferation ability were in a single distribution and had a smaller area compared to the cells on the coverslips and CS-PEO (Table 3, Figure 5 and Figure 6). Rounded, supposably apoptotic, cells were numerous. Cell colonies were not clearly visualized.

Thus, CS-PEO demonstrated satisfactory biocompatibility properties in the in vitro tests with rat MMSC, whereas CS-PEO-CeONP had decreased properties with a lower level of adhered cells and a lower level of cell proliferation. Nevertheless, the latter did not demonstrate apparent cytotoxicity and only decreased biocompatibility compared to CS-PEO.

### 3.6. In Vivo Biocompatibility 

**Figure 7:**
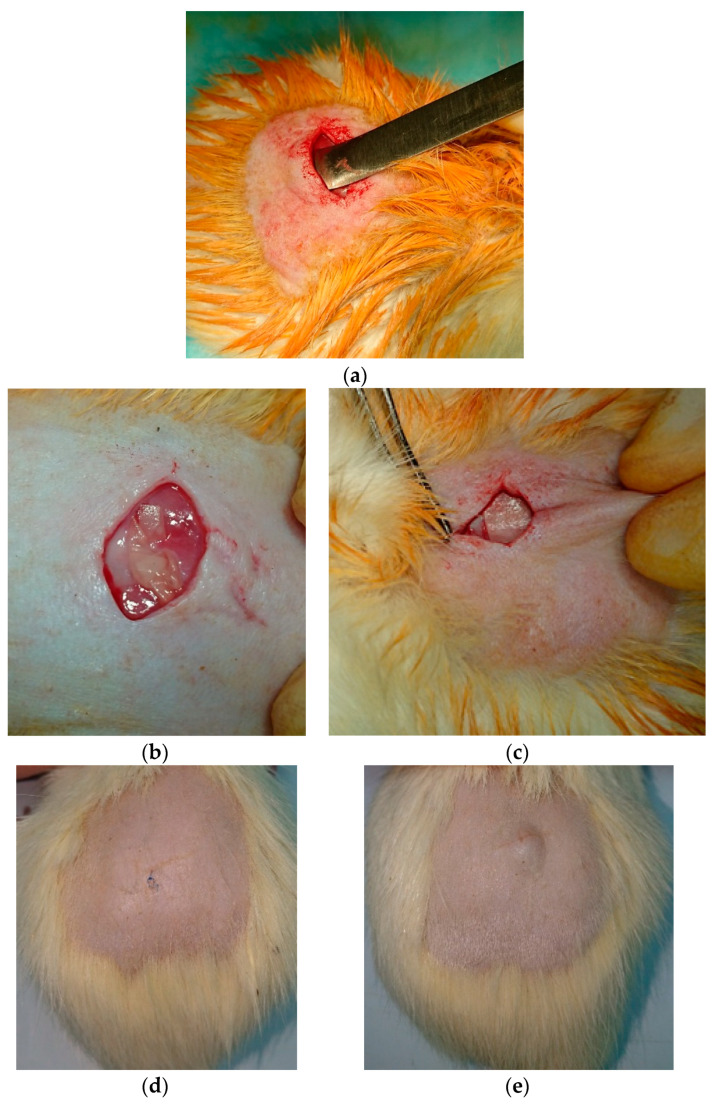
Creation of an implant pocket (**a**), CS-PEO in the implant bed (**b**), CS-PEO-CeONP in the implant bed (**c**), implantation zones on day 14 after implantation: sham-operated group (**d**), and CS-PEO (**e**).

In addition to cytocompatibility, a subcutaneous implantation study was performed to evaluate the degradation and biocompatibility of the materials in vivo (Figure 7a,b). Wound healing was achieved by primary intentional closure. There was no evidence of chronic pain, suture failure, purulent complications, or gross postoperative scarring. Macroscopically, the skin in the implantation zone during the experiment was indistinguishable from the skin in the control and sham-operated groups (Figure 7c,d).

Tissue swelling and formation of a post-implant papule due to swelling of the implant material was observed on day 3 after surgery. It reached its maximum on day 7 after implantation. On day 14, the papule was significantly reduced without rough skin scarring (Figure 7c,d).

**Figure 8:**
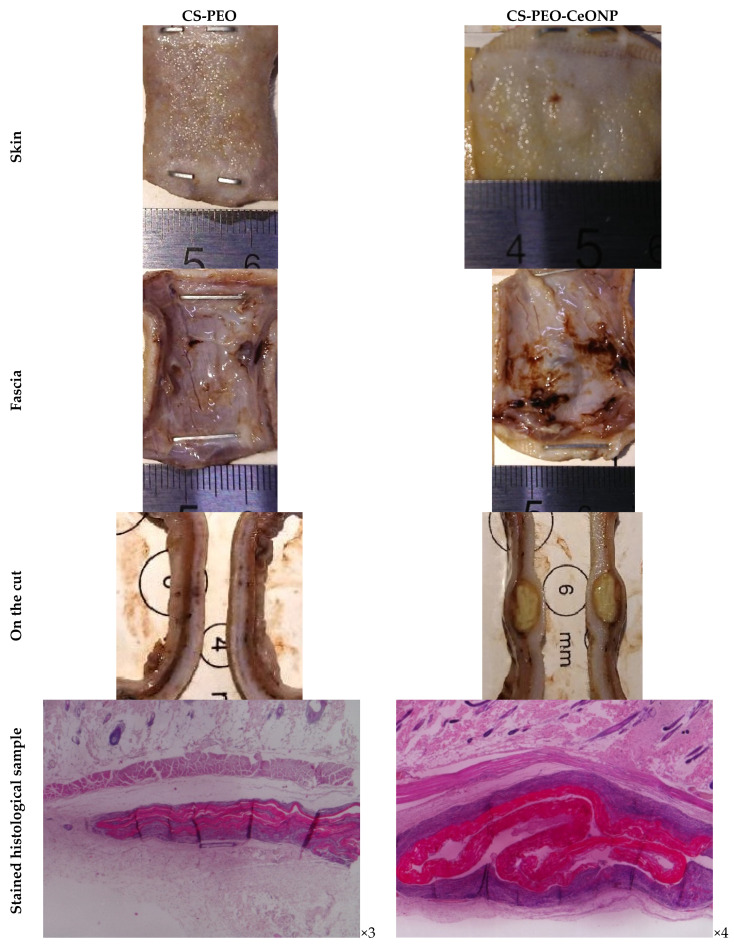
Images of the surgical site and implanted samples through day 30 of the experiment (microscopy in formalin and hematoxylin and eosin).

Macroscopic images of the formalin-fixed specimens at 30 days after surgery are shown in Figure 8. Macroscopically, both matrices were compressed by the tissue. After implantation, CS-PEO-CeONP was preserved and arranged in the implant pocket as a clump, whereas CS-PEO was almost completely destroyed and preserved as layered, sharply thinned fragments.

**Figure 9:**
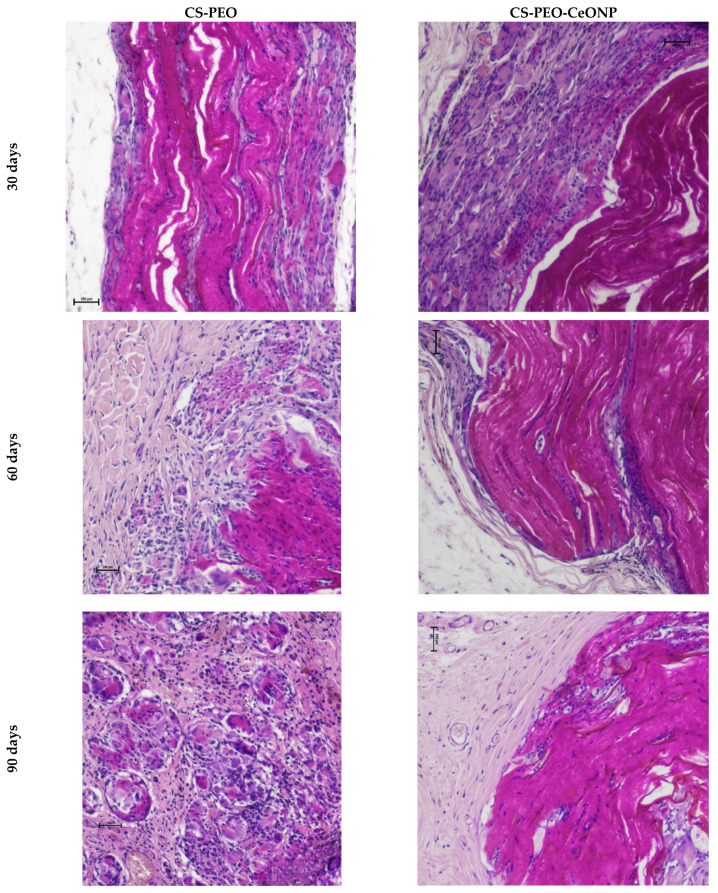
Histology images of implant margins, ×100 (bar 100 μm).

Most of CS-PEO was degraded over the entire thickness after 30 days. The remaining fragments were significantly swollen and cellularly infiltrated (Figure 9). The swollen matrix disintegrated into layers throughout the thickness of the matrix. The remaining material had a heterogeneous structure consisting of dispersed fibrous masses and dense linear fragments, varying in thickness from 350 to 1100 μm. A pronounced reactive cellular infiltration was located at a considerable distance from the matrix fragments and had the character of an aseptic inflammation, represented by macrophages and monocytes, lymphocytes, and multinucleated foreign body-type giant cells (FBGCs). The latter surrounded the rest of the densest material. Macrophages and monocytes infiltrated the spaces between the matrix fragments. Cells penetrated the entire thickness of the matrix, indicating intense bioresorption. Fibroblasts and collagen fibers appeared in areas of resolving inflammation. Pronounced vascularization was observed, with numerous blood vessels in the area of inflammation.

After 60 days, CS-PEO was significantly degraded. Due to swelling, the matrix fragments reached a thickness of 700–1100 μm and were completely infiltrated by cells. Cellular infiltration persisted, but to a lesser extent: the area of inflammation narrowed around the matrix fragments and contained single FBGCs. As the inflammation subsided, the number of fibroblasts and fibrocytes increased, while capsules were not formed. After 90 days, the remaining small matrix fragments were surrounded by macrophages and single FBGCs. The formation of connective tissue around the implant was not observed. 

After 30 days, CS-PEO-CeONP in the implant pocket was compressed by the tissue (Figure 9). Destruction occurred by delamination into fibers and “erosion” of the surface. The material swelled moderately; the thickness of the matrix was 380–400 μm. Reactive cellular infiltration was also pronounced, accompanied by FBGCs forming the peri-implant shaft. After 60 days, the matrix became more optically transparent, and its thickness increased to 550–650 μm. Delamination continued up to half of the matrix’s thickness. Aseptic inflammation decreased compared to the 30-day period. The number of FBGCs was low. Macrophages infiltrated the implant. The formation of a connective tissue capsule and its vascularization were observed. After 90 days, more than half of the matrix remained intact with a thickness of 400–650 μm. The histologic picture after 60 days was similar to the previous one. However, the cell population decreased, and the inflammation disappeared almost completely. The connective tissue capsule was clearly defined. 

Thus, two samples showed different swelling and biodegradation behavior. The degradation of the samples was proceeded by intense swelling, and it was significantly more pronounced for CS-PEO and moderate for CS-PEO-CeONP. Macroscopically and tactilely, CS-PEO was completely degraded and was no longer detectable on day 30. The addition of CeONPs slowed the bioresorption of the CS-PEO base, allowing the matrix to remain almost intact in the tissue up to 90 days after implantation. The incorporation of CeONPs reduces the swelling of the matrix and decreases the intensity of its degradation, which occurs via the delamination of surface layers (for CS-PEO). The CeONP nanofiller leads to matrix degradation by surface erosion without significant swelling of the material. Modification of CS-PEO with CeONPs induces active chronic aseptic inflammation, which differs from CS-PEO in the involvement of FBGCs. This type of infiltration becomes a histological marker of bioresorption, which slows down and disappears with the formation of a peri-implant connective tissue capsule. 

The prolonged period of CS-PEO-CeONP bioresorption may be related to the inhibitory effect of CeONPs on different enzymes involved in CS degradation. CeONPs have been shown to adsorb proteins to their surface, causing unfolding and deactivation of the enzymes of phagocytic cells [30,31]. In this case, changing the concentration of CeONPs in the matrix should control its bioresorption time. In addition, reducing the swelling of the composite fiber mat and the appearance of an insulating connective tissue capsule could prevent the penetration of a large number of cells into the matrix and slow down bioresorption.

## 4. Discussion

The wound healing process is very important for tissue regeneration and for the development of adequate medical systems capable of promoting it. To achieve their therapeutic goals, wound dressings must meet the following requirements: good histocompatibility that does not cause toxicity, inflammation, or immune response [32]; moisture retention and exudate absorption [33]; mechanical properties similar to human skin that maintain its integrity [34,35]; protection against secondary infection [36]; appropriate pore density that allows air permeability [37]; and low adhesion that prevents wound trauma during dressing removal [32].

CS is a well-known substrate for the development of scaffolds for many applications related to regenerative processes and tissue engineering. Due to its biological properties, it is applicable to the design of wound healing materials. For example, nanofibrous CS scaffolds exhibited good gas permeation, high porosity, and high surface area [38]. In addition, CS is able to improve the network structure of wound tissue, influence collagen synthesis and wound tensile strength, and activate macrophages and promote the wound healing process [32,39]. Thus, even unmodified CS with good biocompatibility and biodegradability is able to promote tissue regeneration and collagen fiber growth and can be used as a base material for the development of wound healing scaffolds [32,40]. The use of CS derivatives results in exudate removal, moisture retention, skin regeneration, cell respiration, and hemostasis [38,41,42], and they also exhibit good biocompatible properties.

CeONPs have been shown to possess several biological properties that are critical for the development of healing materials. CeONPs are able to influence the inflammatory process, oxidative stress, angiogenesis, and reduce the risk of infection [43,44]. CeONPs have demonstrated antioxidant activity by scavenging reactive oxygen species and reactive nitrogen species [43,45,46]. CeONPs can activate the Nrf2 signaling pathway and enhance the production and release of antioxidants [47]. Excellent antioxidant activity is also reflected by the magnitude of anti-inflammatory effects. CeONPs are able to activate the NF-κB signaling pathway, which leads to the production of inflammatory cytokines [44]. Meanwhile, treatment with CeONPs reduces the pro-inflammatory activity of endothelial cells, promotes M2 polarization of macrophages, and reduces M1 polarization [48]. Injection of CeONPs causes the inhibition of several pro-inflammatory cytokines and pro-angiogenic growth factors, including *Tslp*, *Lif*, *Il3*, *Il7*, *Vegfa*, *Fgf1*, *Fgf2*, *Fgf7*, *Egf*, *Efna3*, *Lep* [49]. Such an unbalanced immune response is directed against anti-inflammatory activity as well as fibrosis and scar formation. CeONPs can induce angiogenesis by decreasing intracellular oxygen levels [50]. CeONPs regulate several signaling pathways, particularly PI3K/Akt, ERK/MAPK, and Wnt/β-catenin. All are capable of activating cell proliferation and growth [44,45]. CeONPs can stimulate the angiogenesis and proliferation of endothelial cells, thereby increasing the supply of nutrients to the wound site [44,51].

The use of CeONPs can lead to a reduction in intracellular oxygen levels, which induces the translocation of hypoxia-inducing factor 1α (HIF-1α) to the nucleus, activation of the Ref-1/APEI signaling pathway, and, in turn, an increase in several proteins involved in angiogenesis [43,44,52,53]. However, it should be noted that CeONPs may also exhibit anti-angiogenic properties that are microenvironment-dependent. In particular, in an acidic environment, the particles are able to promote hydrogen peroxide accumulation, which in turn could potentially inhibit blood vessel formation. This occurs, for example, in tumor growth [54]. The antibacterial properties of ceria are very promising for use as a long-lasting biocide to prevent bacterial infection, especially in wound healing. The primary antibacterial effect is related to a direct contact with bacterial membranes [55]. Positively charged particles are adsorbed onto bacterial membranes, leading to viscosity changes, disruption of ionic pump function, and disruption of transport between the bacterial cell and the environment [56,57]. CeONPs could negatively affect the function of proteins on the outer membranes of bacteria [58]. In addition, the particles can cause physical damage to bacterial membranes [59]. In some cases, CeONPs can promote an increase in reactive oxygen species production in bacteria, which can cause damage to DNA, RNA, proteins, etc. [57]. 

Thus, both CS and CeONPs exhibit numerous properties that may be influential in the development of scaffolds for wound healing. The use of their strong properties and advantages for this purpose seems very justified.

In this work, the electrospun CS-PEO mats reinforced with CeONPs were obtained. The addition of CeONPs reduced the average fiber diameter in electrospinning, decreased the fiber swelling capacity, increased the mechanical strength, and influenced the biological activity of CS-PEO. Despite the reduced cell adhesion on CS-PEO-CeONP compared to CS-PEO in the in vitro tests, biocompatibility properties were not dramatically reduced, and the samples were suitable for further in vivo testing. In vivo studies showed significantly longer biodegradation of CS-PEO-CeONP compared to CS-PEO. Bioresorption was observed to occur through the intense swelling of the matrices, which was more intense for CS-PEO and moderate for CS-PEO-CeONP. During implantation, both samples showed the development of reactive aseptic inflammation, accompanied by multinucleated FBGCs. For CS-PEO-CeONP, this inflammation was resolved by day 90 of the experiment, with the matrix still present in the tissue. A reduction in inflammation during the observation period occurred in both samples, but a peri-implant capsule around the preserved matrix was observed only in the CeONP-doped sample. Thus, CeONPs can control the swelling and bioresorption rate of electrospun mats.

In addition, CeONPs help to reduce the inflammatory response and stimulate the growth of an ordered layer of connective tissue near the matrix. This biocompatibility result further slows the biodegradation of the matrix (making it tunable) and, most importantly, allows for the formation of a vascular bed. It is believed that such a material placed on the surface of the wound will act as a dressing, isolate the area of injury from infectious factors, prevent dehydration, and (through the effect of the crust) stimulate the growth of a connective tissue plate, supporting the vascular bed and epithelial proliferation. Our results suggest that the resulting CS-PEO-CeONP hybrid material may have promising applications in regenerative medicine, including the healing of extensive skin wounds or burns and the closure of large post-traumatic defects. Moderate swelling, the delayed and regulated bioresorption of the matrix, and its elasticity allow this material to be considered in the design of devices for the closure of special wound surfaces, such as the eyeball, brain, tympanic membrane, and others.

## Figures and Tables

**Table 1:** Mechanical properties of the electrospun mats.

Sample	E (MPa)	σ_y_ (MPa)	σ_b_ (MPa)	ε_b_ (%)
CS-PEO	455 ± 46	12 ± 1	13 ± 2	6.2 ± 0.7
CS-PEO-CeONP	689 ± 29	14 ± 1	15 ± 1	5.4 ± 0.4

**Table 2:** Swelling of nonwoven materials.

Sample	Swelling in Water, g/g	Swelling in 0.9% NaCl, g/g
CS-PEO	3.3	3.1
CS-PEO-CeONP	5.2	3.8

**Table 3:** Description of the adhered cells and cell colonies on the samples.

Sample	Single Cells		Type of Cell Colonies	Adhered MMSC, Cells/mm^2^
	Elongated	Round		
Glass	multiple	-	Plane colony/monolayer	-
CS-PEO	multiple	multiple	Individual cells	80 ± 4
CS-PEO-CeONP	single	multiple	Individual cells	83 ± 5

## Data Availability

Data are contained within this article and are available upon request.
